# Crystal structure of di­chlorido­{2,6-bis­[(3-phenyl-1*H*-pyrazol-1-yl)meth­yl]pyridine}cobalt(II)

**DOI:** 10.1107/S2056989015003862

**Published:** 2015-03-04

**Authors:** Kyung-sun Son, Jeong Oh Woo, Daeyoung Kim, Sung Kwon Kang

**Affiliations:** a Department of Chemistry, Chungnam National University, Daejeon 305-764, Republic of Korea

**Keywords:** crystal structure, Co^II^ complex, pyrazolylpyrid­yl, C—H ⋯π inter­actions, π–π inter­actions

## Abstract

In the title complex, [CoCl_2_(C_25_H_21_N_5_)], the Co^II^ atom is coordinated by two Cl atoms and two N atoms, provided by a tridentate pyrazolylpyridyl ligand, forming a slightly distorted tetra­hedral geometry [range of angles: 96.51 (10) (chelate ring) to 118.60 (9)°]. The dihedral angle between Cl/Co/Cl and N/Co/N planes is 86.83 (7)°. The chelate ring has the conformation of a distorted boat. The dihedral angle between pyridyl ring and the coordinated pyrazolyl ring is 56.16 (12)°. The uncoordinated pyrazolyl ring is almost perpendicular to the pyridyl ring with the dihedral angle of 87.49 (10)°. In the crystal packing, inter­molecular phenyl-C—H ⋯π(pyrid­yl) inter­actions generate dimeric aggregates. These are connected into a zigzag supra­molecular chain along the *c*-axis direction *via* π–π inter­actions [inter-centroid distance between pyridyl and phenyl rings = 3.664 (2) Å].

## Related literature   

For the synthesis of the title compound, see: Reger *et al.* (2005 ▸); Son *et al.* (2014 ▸). For metal complexes with similar ligands, see: Massoud *et al.* (2013 ▸); Sharma *et al.* (2011 ▸); Ojwach *et al.* (2007 ▸); Manikandan *et al.* (2000 ▸, 2001 ▸); Halcrow & Kilner (2002 ▸); Foster *et al.* (2002 ▸). For the potential applications of the ligand in catalysis, see: Karam *et al.* (2005 ▸).
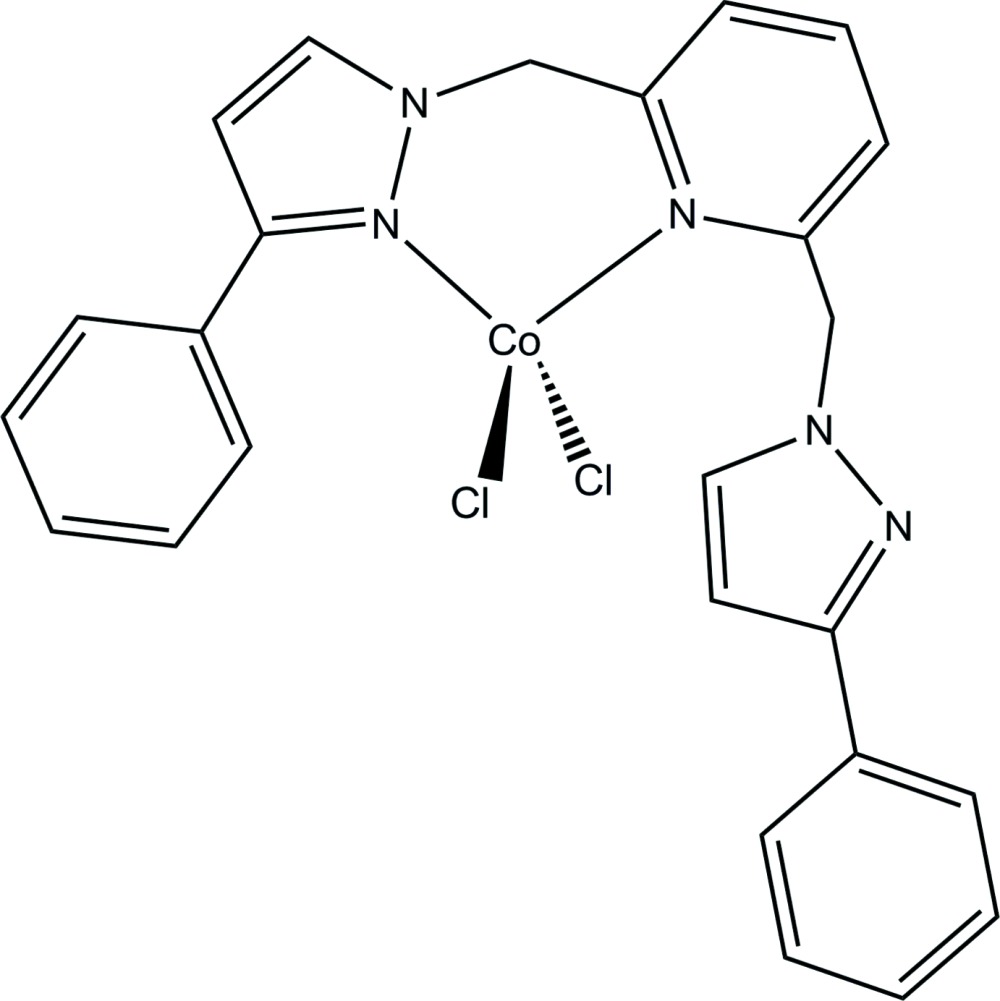


## Experimental   

### Crystal data  

[CoCl_2_(C_25_H_21_N_5_)]
*M* *_r_* = 521.3
Monoclinic, 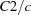

*a* = 12.9766 (4) Å
*b* = 10.5867 (3) Å
*c* = 33.5943 (9) Å
β = 93.2592 (19)°
*V* = 4607.7 (2) Å^3^
*Z* = 8
Mo *K*α radiation
μ = 1.00 mm^−1^
*T* = 296 K
0.25 × 0.22 × 0.1 mm

### Data collection  

Bruker SMART CCD area-detector diffractometer
Absorption correction: multi-scan (*SADABS*; Bruker, 2002 ▸) *T*_min_ = 0.76, *T*_max_ = 0.903
25697 measured reflections
5718 independent reflections
3751 reflections with *I* > 2σ(*I*)
*R*_int_ = 0.072

### Refinement  

*R*[*F* ^2^ > 2σ(*F* ^2^)] = 0.058
*wR*(*F* ^2^) = 0.132
*S* = 1.06
5718 reflections
298 parameters
H-atom parameters constrained
Δρ_max_ = 0.52 e Å^−3^
Δρ_min_ = −0.47 e Å^−3^

### 

Data collection: *SMART* (Bruker, 2002 ▸); cell refinement: *SAINT* (Bruker, 2002 ▸); data reduction: *SAINT*; program(s) used to solve structure: *SHELXS2013* (Sheldrick, 2008 ▸); program(s) used to refine structure: *SHELXL2013* (Sheldrick, 2015 ▸); molecular graphics: *ORTEP-3 for Windows* (Farrugia, 2012 ▸); software used to prepare material for publication: *WinGX* (Farrugia, 2012 ▸).

## Supplementary Material

Crystal structure: contains datablock(s) global, I. DOI: 10.1107/S2056989015003862/tk5360sup1.cif

Structure factors: contains datablock(s) I. DOI: 10.1107/S2056989015003862/tk5360Isup2.hkl

**Fig. 1.**
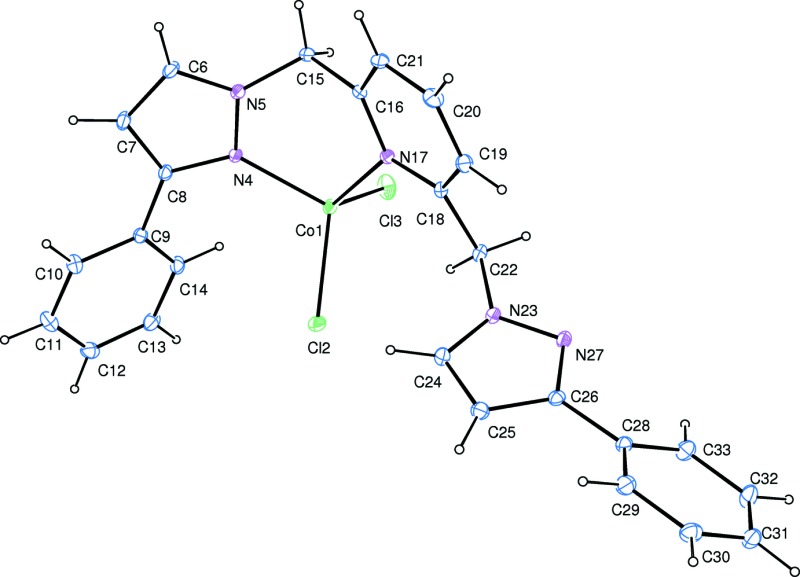
Molecular structure of the title compound, showing the atom-numbering scheme and 30% probability ellipsoids.

**Fig. 2.**
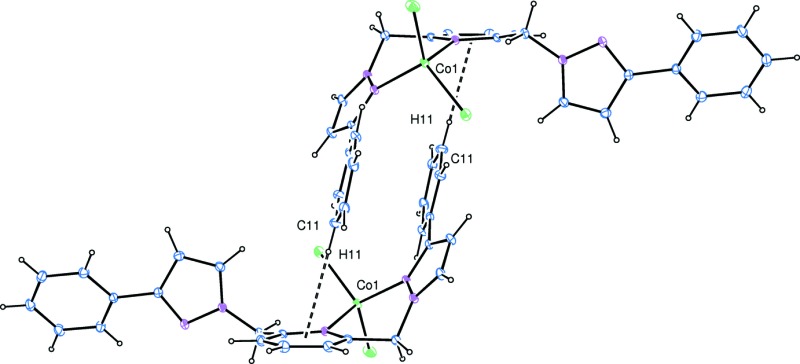
Dimer formation via C—H···π interactions.

. DOI: 10.1107/S2056989015003862/tk5360fig1.tif
Mol­ecular structure of the title compound, showing the atom-numbering scheme and 30% probability ellipsoids.

via . DOI: 10.1107/S2056989015003862/tk5360fig2.tif
Dimer formation *via* C—H⋯π inter­actions.

CCDC reference: 1051138

Additional supporting information:  crystallographic information; 3D view; checkCIF report

## Figures and Tables

**Table 1:** Selected bond lengths ()

Co1N4	2.041(3)
Co1N17	2.109(3)
Co1Cl3	2.2030(11)
Co1Cl2	2.2499(10)

**Table 2:** Hydrogen-bond geometry (, )

*D*H*A*	*D*H	H*A*	*D* *A*	*D*H*A*
C11H11*Cg*1^i^	0.93	2.87	3.806(4)	180

Symmetry code: (i) 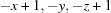.

## supplementary crystallographic information

### S1. Experimental

To a stirred solution of 2,6-pyridinedimethanol (0.28 g, 2.0 mmol) and NaOH (0.8 g, 20 mmol) in tetrahydrofuran (THF)/water (7.5/7.5 ml) was added a solution of *p*-toluenesulfonyl chloride (0.76 g, 4.0 mmol) in THF (7.5 ml) at 0 °C. After 4 h of stirring, the mixture was poured into 20 ml of water and extracted with methylene chloride three times. The organic layer was washed with saturated aqueous NaCl solution and distilled water, and dried over Na_2_SO_4_; the solvent was removed *in vacuo* to afford 2,6-pyridine-dimethylene-ditosylate (0.788 g, 88%) as a white powder. In a separate flask under a nitrogen atmosphere, a solution of 3-phenyl-1*H*-pyrazole (0.61 g, 5.34 mmol) in dry THF (10 ml) was added drop-wise to a suspension of NaH (0.13 g, 5.34 mmol) in dry THF (10 ml) at 0 °C. After 15 min of stirring, a solution of 2,6-pyridine-dimethylene-ditosylate (1.20 g, 2.67 mmol) in dry THF (15 ml) was added to this solution; the mixture was stirred overnight, filtered, and the solvent was removed. The crude product was purified by column chromatography on silica gel with ethyl acetate : hexane = 1:1 as eluent to afford 0.41 g (40%) of pure ligand as a white oil.

To a solution of CoCl_2_ (2.6 mg, 0.02 mmol) in THF (2 ml) was added a solution of the organic ligand (7.8 mg, 0.02 mmol) in THF (2 ml) drop-wise at 40 °C. The solution was stirred vigorously. A deep-blue suspension was formed immediately. The product was isolated as a blue powder by removing the solvent, washed repeatedly with THF followed by diethyl ether, and dried *in vacuo*. Deep-blue single crystals of the title compound were obtained by slow evaporation of its concentrated solution in dichloromethane at room temperature.

### S2. Refinement

All H atoms were positioned geometrically and refined using a riding model, with C—H = 0.93–0.97 Å, and with U_iso_(H) = 1.2U_eq_(C).

### Crystal data

**Table:**

[CoCl_2_(C_25_H_21_N_5_)]	*F*(000) = 2136
*M**_r_* = 521.3	*D*_x_ = 1.503 Mg m^−^^3^
Monoclinic, *C*2/*c*	Mo *K*α radiation, λ = 0.71073 Å
Hall symbol: -C 2yc	Cell parameters from 4379 reflections
*a* = 12.9766 (4) Å	θ = 2.4–23.4°
*b* = 10.5867 (3) Å	µ = 1.00 mm^−^^1^
*c* = 33.5943 (9) Å	*T* = 296 K
β = 93.2592 (19)°	Plate, deep-blue
*V* = 4607.7 (2) Å^3^	0.25 × 0.22 × 0.1 mm
*Z* = 8	

### Data collection

**Table:**

Bruker SMART CCD area-detector diffractometer	3751 reflections with *I* > 2σ(*I*)
Radiation source: fine-focus sealed tube	*R*_int_ = 0.072
φ and ω scans	θ_max_ = 28.3°, θ_min_ = 2.4°
Absorption correction: multi-scan (*SADABS*; Bruker, 2002)	*h* = −15→17
*T*_min_ = 0.76, *T*_max_ = 0.903	*k* = −14→14
25697 measured reflections	*l* = −44→44
5718 independent reflections	

### Refinement

**Table:**

Refinement on *F*^2^	0 restraints
Least-squares matrix: full	Hydrogen site location: inferred from neighbouring sites
*R*[*F*^2^ > 2σ(*F*^2^)] = 0.058	H-atom parameters constrained
*wR*(*F*^2^) = 0.132	*w* = 1/[σ^2^(*F*_o_^2^) + (0.048*P*)^2^ + 4.3485*P*] where *P* = (*F*_o_^2^ + 2*F*_c_^2^)/3
*S* = 1.06	(Δ/σ)_max_ = 0.002
5718 reflections	Δρ_max_ = 0.52 e Å^−^^3^
298 parameters	Δρ_min_ = −0.47 e Å^−^^3^

### Special details

**Table:**

Geometry. All e.s.d.'s (except the e.s.d. in the dihedral angle between two l.s. planes) are estimated using the full covariance matrix. The cell e.s.d.'s are taken into account individually in the estimation of e.s.d.'s in distances, angles and torsion angles; correlations between e.s.d.'s in cell parameters are only used when they are defined by crystal symmetry. An approximate (isotropic) treatment of cell e.s.d.'s is used for estimating e.s.d.'s involving l.s. planes.

### Fractional atomic coordinates and isotropic or equivalent isotropic displacement parameters (Å^2^)

**Table:**

	*x*	*y*	*z*	*U*_iso_*/*U*_eq_	
Co1	0.39083 (3)	0.22404 (4)	0.58917 (2)	0.03468 (14)	
Cl2	0.56212 (7)	0.23374 (10)	0.58231 (3)	0.0500 (2)	
Cl3	0.32167 (10)	0.40748 (10)	0.60338 (3)	0.0672 (3)	
N4	0.3229 (2)	0.1187 (3)	0.54406 (7)	0.0331 (6)	
N5	0.2543 (2)	0.0299 (3)	0.55511 (7)	0.0366 (6)	
C6	0.2331 (3)	−0.0533 (3)	0.52567 (10)	0.0443 (9)	
H6	0.1887	−0.122	0.5266	0.053*	
C7	0.2884 (3)	−0.0185 (3)	0.49419 (10)	0.0436 (9)	
H7	0.2894	−0.059	0.4696	0.052*	
C8	0.3435 (3)	0.0901 (3)	0.50604 (9)	0.0347 (7)	
C9	0.4137 (2)	0.1637 (3)	0.48268 (9)	0.0342 (7)	
C10	0.4601 (3)	0.1057 (4)	0.45118 (10)	0.0475 (9)	
H10	0.4465	0.021	0.4457	0.057*	
C11	0.5260 (3)	0.1714 (4)	0.42791 (12)	0.0562 (11)	
H11	0.5554	0.1316	0.4066	0.067*	
C12	0.5480 (3)	0.2960 (4)	0.43629 (11)	0.0534 (11)	
H12	0.5953	0.3394	0.4216	0.064*	
C13	0.5003 (3)	0.3558 (4)	0.46622 (11)	0.0509 (10)	
H13	0.513	0.4411	0.471	0.061*	
C14	0.4333 (3)	0.2909 (4)	0.48940 (10)	0.0436 (9)	
H14	0.4012	0.3328	0.5096	0.052*	
C15	0.2098 (3)	0.0399 (4)	0.59390 (9)	0.0403 (8)	
H15A	0.1516	−0.0174	0.5947	0.048*	
H15B	0.1841	0.1251	0.5972	0.048*	
C16	0.2864 (2)	0.0096 (3)	0.62810 (9)	0.0333 (7)	
N17	0.3619 (2)	0.0971 (2)	0.63560 (7)	0.0314 (6)	
C18	0.4258 (2)	0.0796 (3)	0.66823 (9)	0.0330 (7)	
C19	0.4218 (3)	−0.0281 (4)	0.69132 (10)	0.0430 (9)	
H19	0.4689	−0.0395	0.7129	0.052*	
C20	0.3486 (3)	−0.1176 (4)	0.68232 (11)	0.0492 (10)	
H20	0.346	−0.1911	0.6974	0.059*	
C21	0.2784 (3)	−0.0975 (4)	0.65052 (10)	0.0438 (9)	
H21	0.2264	−0.156	0.6444	0.053*	
C22	0.4977 (3)	0.1871 (3)	0.68058 (10)	0.0364 (8)	
H22A	0.5015	0.2456	0.6585	0.044*	
H22B	0.4696	0.2324	0.7026	0.044*	
N23	0.6008 (2)	0.1435 (3)	0.69249 (8)	0.0363 (6)	
C24	0.6654 (3)	0.0790 (3)	0.67056 (10)	0.0420 (8)	
H24	0.6533	0.0544	0.6441	0.05*	
C25	0.7519 (3)	0.0558 (3)	0.69398 (10)	0.0436 (9)	
H25	0.8105	0.0122	0.6871	0.052*	
C26	0.7342 (3)	0.1112 (3)	0.73064 (9)	0.0352 (7)	
N27	0.6417 (2)	0.1659 (3)	0.72992 (8)	0.0374 (7)	
C28	0.8065 (3)	0.1190 (3)	0.76634 (10)	0.0376 (8)	
C29	0.8786 (3)	0.0243 (4)	0.77423 (12)	0.0503 (9)	
H29	0.8804	−0.0454	0.7574	0.06*	
C30	0.9484 (3)	0.0329 (4)	0.80719 (12)	0.0550 (10)	
H30	0.9961	−0.0314	0.8124	0.066*	
C31	0.9473 (3)	0.1347 (4)	0.83167 (11)	0.0557 (11)	
H31	0.9954	0.1413	0.8532	0.067*	
C32	0.8750 (3)	0.2281 (4)	0.82467 (12)	0.0586 (11)	
H32	0.873	0.2968	0.8419	0.07*	
C33	0.8047 (3)	0.2202 (4)	0.79195 (11)	0.0493 (9)	
H33	0.756	0.2838	0.7874	0.059*	

### Atomic displacement parameters (Å^2^)

**Table:**

	*U*^11^	*U*^22^	*U*^33^	*U*^12^	*U*^13^	*U*^23^
Co1	0.0414 (3)	0.0335 (3)	0.0290 (2)	−0.0090 (2)	0.00033 (17)	−0.00083 (19)
Cl2	0.0439 (5)	0.0617 (6)	0.0444 (5)	−0.0201 (5)	0.0022 (4)	0.0001 (4)
Cl3	0.1011 (9)	0.0450 (6)	0.0563 (6)	0.0149 (6)	0.0115 (6)	−0.0023 (5)
N4	0.0346 (15)	0.0383 (16)	0.0263 (13)	−0.0087 (12)	0.0011 (11)	−0.0016 (11)
N5	0.0377 (16)	0.0422 (17)	0.0297 (13)	−0.0109 (13)	0.0008 (11)	−0.0011 (12)
C6	0.050 (2)	0.043 (2)	0.0392 (19)	−0.0181 (18)	−0.0066 (16)	−0.0034 (16)
C7	0.056 (2)	0.043 (2)	0.0309 (17)	−0.0089 (18)	−0.0021 (15)	−0.0078 (15)
C8	0.0377 (18)	0.039 (2)	0.0267 (15)	−0.0016 (15)	−0.0035 (13)	−0.0009 (14)
C9	0.0342 (18)	0.040 (2)	0.0279 (15)	0.0005 (15)	−0.0028 (13)	0.0022 (14)
C10	0.057 (2)	0.045 (2)	0.0408 (19)	0.0037 (19)	0.0075 (17)	−0.0028 (17)
C11	0.059 (3)	0.065 (3)	0.046 (2)	0.011 (2)	0.0181 (19)	0.003 (2)
C12	0.045 (2)	0.076 (3)	0.039 (2)	−0.006 (2)	0.0033 (16)	0.017 (2)
C13	0.061 (3)	0.050 (2)	0.040 (2)	−0.018 (2)	−0.0066 (18)	0.0047 (17)
C14	0.055 (2)	0.047 (2)	0.0291 (16)	−0.0053 (18)	0.0015 (15)	0.0001 (15)
C15	0.0321 (18)	0.056 (2)	0.0334 (17)	−0.0123 (17)	0.0030 (13)	0.0007 (16)
C16	0.0312 (17)	0.0408 (19)	0.0287 (15)	−0.0066 (15)	0.0079 (13)	−0.0017 (14)
N17	0.0319 (14)	0.0340 (15)	0.0284 (13)	−0.0035 (12)	0.0016 (11)	0.0011 (11)
C18	0.0361 (18)	0.0368 (19)	0.0264 (15)	−0.0012 (15)	0.0028 (13)	−0.0031 (13)
C19	0.046 (2)	0.050 (2)	0.0329 (17)	−0.0017 (18)	0.0001 (15)	0.0083 (16)
C20	0.058 (3)	0.041 (2)	0.049 (2)	−0.0077 (19)	0.0103 (18)	0.0156 (17)
C21	0.042 (2)	0.047 (2)	0.0427 (19)	−0.0185 (17)	0.0074 (16)	0.0024 (16)
C22	0.0421 (19)	0.0361 (19)	0.0301 (16)	−0.0028 (15)	−0.0057 (14)	−0.0019 (13)
N23	0.0362 (16)	0.0415 (17)	0.0309 (14)	−0.0044 (13)	−0.0016 (11)	−0.0028 (12)
C24	0.045 (2)	0.045 (2)	0.0354 (18)	−0.0048 (17)	0.0015 (15)	−0.0069 (16)
C25	0.041 (2)	0.043 (2)	0.047 (2)	−0.0017 (17)	0.0055 (16)	−0.0038 (16)
C26	0.0341 (18)	0.0339 (18)	0.0375 (17)	−0.0085 (15)	−0.0001 (14)	0.0033 (14)
N27	0.0418 (17)	0.0408 (17)	0.0289 (14)	−0.0057 (14)	−0.0035 (12)	0.0003 (12)
C28	0.0342 (19)	0.044 (2)	0.0345 (17)	−0.0067 (16)	0.0000 (14)	0.0042 (15)
C29	0.047 (2)	0.052 (2)	0.052 (2)	−0.0003 (19)	0.0010 (18)	−0.0040 (19)
C30	0.042 (2)	0.064 (3)	0.059 (2)	0.003 (2)	−0.0032 (18)	0.013 (2)
C31	0.050 (2)	0.073 (3)	0.042 (2)	−0.013 (2)	−0.0068 (18)	0.004 (2)
C32	0.067 (3)	0.061 (3)	0.047 (2)	−0.004 (2)	−0.0086 (19)	−0.016 (2)
C33	0.049 (2)	0.046 (2)	0.052 (2)	0.0038 (19)	−0.0074 (17)	0.0010 (18)

### Geometric parameters (Å, º)

**Table:**

Co1—N4	2.041 (3)	C18—C19	1.381 (5)
Co1—N17	2.109 (3)	C18—C22	1.515 (4)
Co1—Cl3	2.2030 (11)	C19—C20	1.364 (5)
Co1—Cl2	2.2499 (10)	C19—H19	0.93
N4—C8	1.354 (4)	C20—C21	1.380 (5)
N4—N5	1.361 (4)	C20—H20	0.93
N5—C6	1.341 (4)	C21—H21	0.93
N5—C15	1.459 (4)	C22—N23	1.450 (4)
C6—C7	1.362 (5)	C22—H22A	0.97
C6—H6	0.93	C22—H22B	0.97
C7—C8	1.400 (5)	N23—C24	1.335 (4)
C7—H7	0.93	N23—N27	1.358 (3)
C8—C9	1.461 (5)	C24—C25	1.356 (5)
C9—C14	1.386 (5)	C24—H24	0.93
C9—C10	1.390 (5)	C25—C26	1.395 (5)
C10—C11	1.378 (5)	C25—H25	0.93
C10—H10	0.93	C26—N27	1.331 (4)
C11—C12	1.376 (6)	C26—C28	1.482 (4)
C11—H11	0.93	C28—C33	1.375 (5)
C12—C13	1.366 (6)	C28—C29	1.388 (5)
C12—H12	0.93	C29—C30	1.393 (5)
C13—C14	1.383 (5)	C29—H29	0.93
C13—H13	0.93	C30—C31	1.356 (6)
C14—H14	0.93	C30—H30	0.93
C15—C16	1.510 (4)	C31—C32	1.375 (6)
C15—H15A	0.97	C31—H31	0.93
C15—H15B	0.97	C32—C33	1.390 (5)
C16—N17	1.361 (4)	C32—H32	0.93
C16—C21	1.369 (5)	C33—H33	0.93
N17—C18	1.349 (4)		
			
N4—Co1—N17	96.51 (10)	C16—N17—Co1	116.9 (2)
N4—Co1—Cl3	118.60 (9)	N17—C18—C19	121.9 (3)
N17—Co1—Cl3	108.05 (8)	N17—C18—C22	117.4 (3)
N4—Co1—Cl2	109.65 (8)	C19—C18—C22	120.6 (3)
N17—Co1—Cl2	108.85 (8)	C20—C19—C18	119.8 (3)
Cl3—Co1—Cl2	113.51 (5)	C20—C19—H19	120.1
C8—N4—N5	105.7 (2)	C18—C19—H19	120.1
C8—N4—Co1	136.3 (2)	C19—C20—C21	119.0 (3)
N5—N4—Co1	115.88 (18)	C19—C20—H20	120.5
C6—N5—N4	111.2 (3)	C21—C20—H20	120.5
C6—N5—C15	129.2 (3)	C16—C21—C20	119.1 (3)
N4—N5—C15	119.5 (3)	C16—C21—H21	120.5
N5—C6—C7	107.5 (3)	C20—C21—H21	120.5
N5—C6—H6	126.3	N23—C22—C18	112.4 (3)
C7—C6—H6	126.3	N23—C22—H22A	109.1
C6—C7—C8	106.5 (3)	C18—C22—H22A	109.1
C6—C7—H7	126.7	N23—C22—H22B	109.1
C8—C7—H7	126.7	C18—C22—H22B	109.1
N4—C8—C7	109.1 (3)	H22A—C22—H22B	107.9
N4—C8—C9	123.4 (3)	C24—N23—N27	112.0 (3)
C7—C8—C9	127.5 (3)	C24—N23—C22	127.5 (3)
C14—C9—C10	118.0 (3)	N27—N23—C22	120.4 (3)
C14—C9—C8	123.0 (3)	N23—C24—C25	107.3 (3)
C10—C9—C8	118.9 (3)	N23—C24—H24	126.3
C11—C10—C9	121.2 (4)	C25—C24—H24	126.3
C11—C10—H10	119.4	C24—C25—C26	105.3 (3)
C9—C10—H10	119.4	C24—C25—H25	127.4
C12—C11—C10	119.7 (4)	C26—C25—H25	127.4
C12—C11—H11	120.1	N27—C26—C25	111.1 (3)
C10—C11—H11	120.1	N27—C26—C28	121.2 (3)
C13—C12—C11	119.8 (4)	C25—C26—C28	127.5 (3)
C13—C12—H12	120.1	C26—N27—N23	104.2 (3)
C11—C12—H12	120.1	C33—C28—C29	118.6 (3)
C12—C13—C14	120.7 (4)	C33—C28—C26	121.2 (3)
C12—C13—H13	119.7	C29—C28—C26	120.2 (3)
C14—C13—H13	119.7	C28—C29—C30	120.4 (4)
C13—C14—C9	120.4 (3)	C28—C29—H29	119.8
C13—C14—H14	119.8	C30—C29—H29	119.8
C9—C14—H14	119.8	C31—C30—C29	120.4 (4)
N5—C15—C16	112.8 (3)	C31—C30—H30	119.8
N5—C15—H15A	109	C29—C30—H30	119.8
C16—C15—H15A	109	C30—C31—C32	119.9 (4)
N5—C15—H15B	109	C30—C31—H31	120
C16—C15—H15B	109	C32—C31—H31	120
H15A—C15—H15B	107.8	C31—C32—C33	120.2 (4)
N17—C16—C21	122.5 (3)	C31—C32—H32	119.9
N17—C16—C15	115.6 (3)	C33—C32—H32	119.9
C21—C16—C15	121.8 (3)	C28—C33—C32	120.5 (4)
C18—N17—C16	117.4 (3)	C28—C33—H33	119.7
C18—N17—Co1	124.2 (2)	C32—C33—H33	119.7

### Hydrogen-bond geometry (Å, º)

**Table:**

*D*—H···*A*	*D*—H	H···*A*	*D*···*A*	*D*—H···*A*
C11—H11···*Cg*1^i^	0.93	2.87	3.806 (4)	180

Symmetry code: (i) −*x*+1, −*y*, −*z*+1.
