# Supplementary figures and images for: Crystal structure of di­chlorido­{2,6-bis­[(3-phenyl-1H-pyrazol-1-yl)meth­yl]pyridine}cobalt(II)
Source: Acta Crystallogr E Crystallogr Commun. 2015 Mar 4;71(Pt 4):m75–6. doi: 10.1107/S2056989015003862 (PMC4438808; doi:10.1107/S2056989015003862)

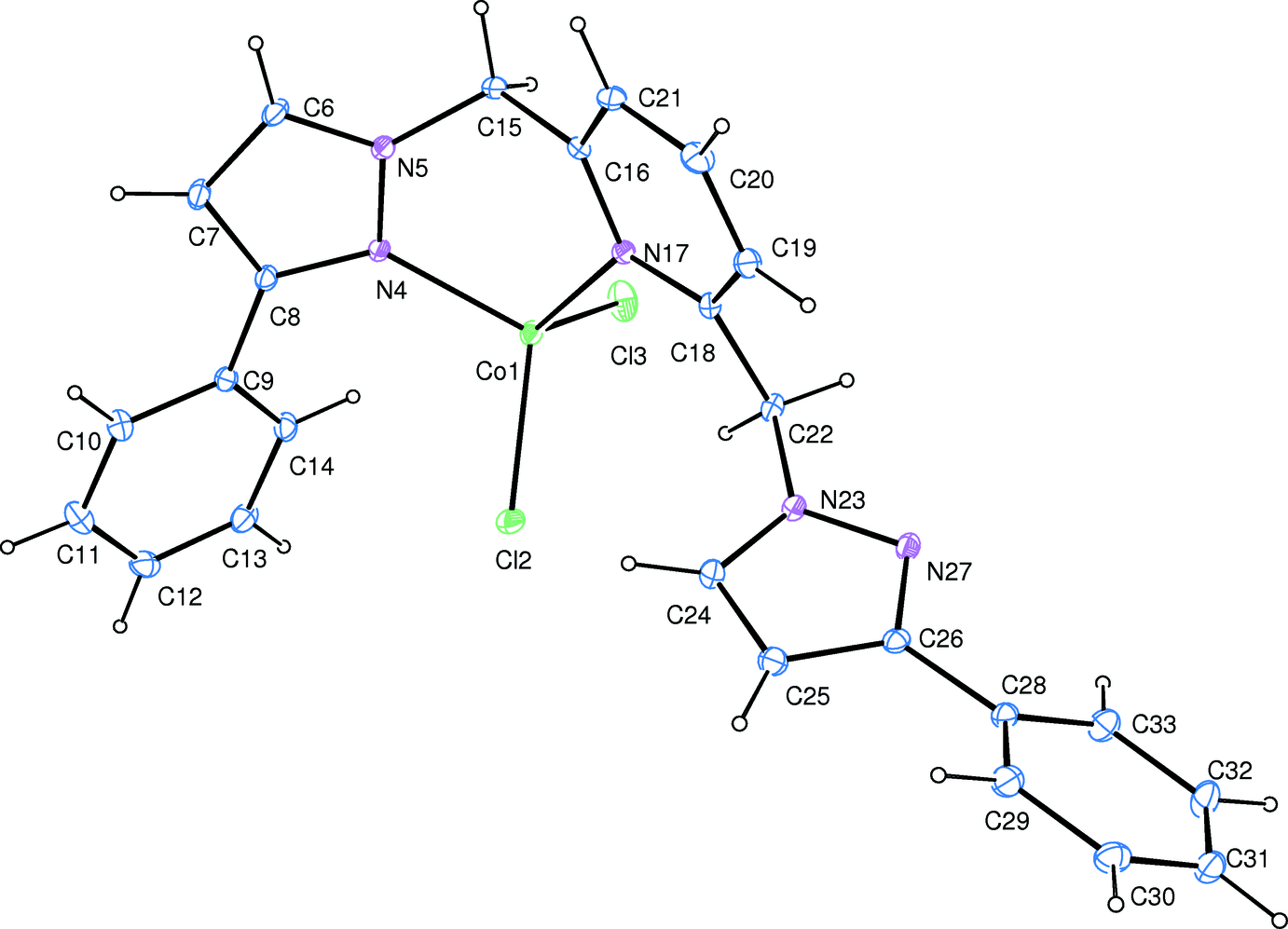

Supplement: Supplementary file 3 [file e-71-00m75-fig1.tif]

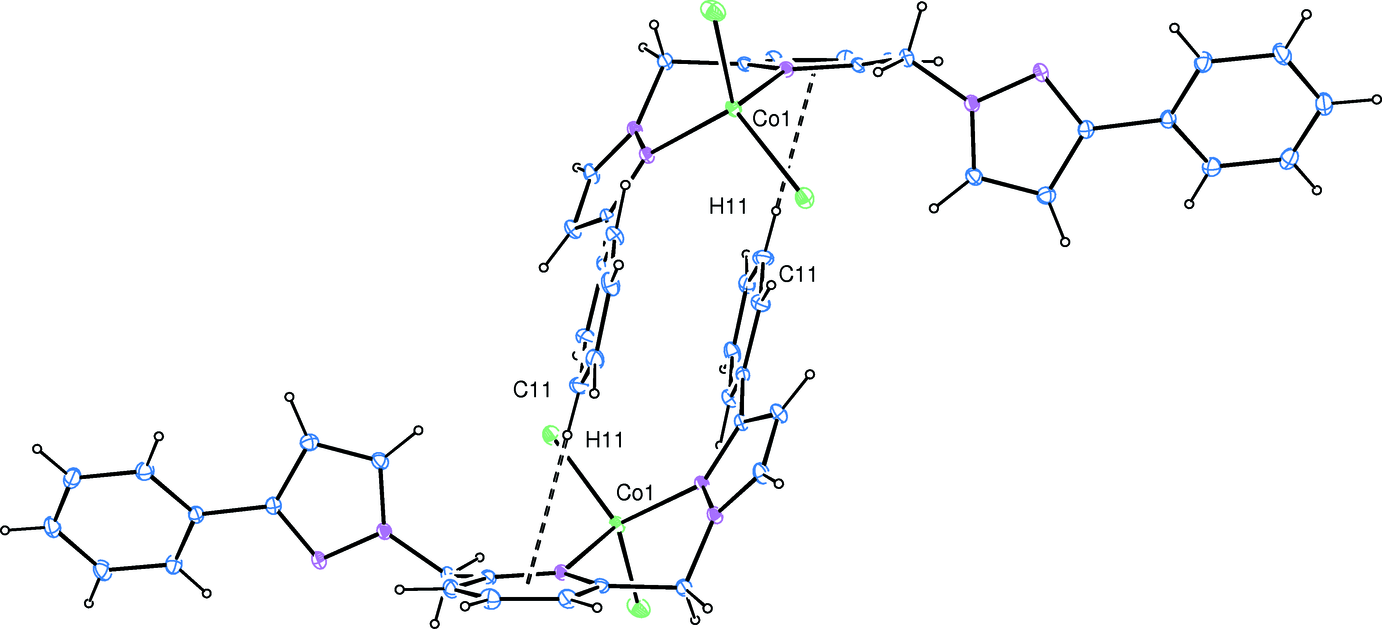

Supplement: Supplementary file 4 [file e-71-00m75-fig2.tif]
